# Effect of salinity on C1-gas fermentation by *Clostridium carboxidivorans* producing acids and alcohols

**DOI:** 10.1186/s13568-019-0837-y

**Published:** 2019-07-17

**Authors:** Ánxela Fernández-Naveira, María C. Veiga, Christian Kennes

**Affiliations:** Chemical Engineering Laboratory, Faculty of Sciences and Center for Advanced Scientific Research (CICA), University of La Coruña, Rúa da Fraga 10, 15008 La Coruña, Spain

**Keywords:** *Clostridium carboxidivorans*, Carbon dioxide, Carbon monoxide, Ethanol, Butanol

## Abstract

*Clostridium carboxidivorans* can produce acids and/or alcohols through syngas fermentation. In that C1-gas fermentation process, the production of acids takes place at higher pH (acetogenesis) (e.g., around 6.00), while the conversion of accumulated acids into alcohols (solventogenesis) is more favourable at a lower pH (e.g., 4.75–5.00). The pH drop, when switching from acetogenesis to solventogenesis, can either be natural—and result from the production of acids—or artificial. In the latter case, for the acidification process, a strong acid (HCl) was added to a syngas fermenting bioreactor in this study, while NaOH was added to increase the pH whenever needed. Cycles of high and low pH were applied in order to switch from acetogenic to solventogenic stages. This pH adjustment procedure leads to the accumulation of salts. The possible inhibitory effect exerted by changes in salinity in the bioreactor was estimated in batch bottles assays, carried out with different salinities (media with different concentrations of sodium chloride) using *C. carboxidivorans* and CO as a carbon source. At NaCl concentrations below 9 g/L, maximum growth rates around 0.055 h^−1^ were obtained, whereas increasing the concentration of sodium chloride had a negative effect on bacterial growth and CO consumption. In the case of the most concentrated bottles, above 15 g/L NaCl no relevant growth was observed. Also, the IC_50_, i.e. concentration yielding 50% growth inhibition, was estimated, and reached a value of 11 g/L sodium chloride.

## Introduction

Biorefineries based on the production of biofuels and platform chemicals from renewable sources, biomass, or wastes represent an environmentally-friendly alternative to more conventional oil refineries and chemical industries. A key challenge in biofuels production is to develop a low cost and effective process, to make such production competitive and viable (Branduardi and Porro 2016). Biorefineries are mainly classified into first and second generation processes. The raw material used in first generation biorefineries has to deal with food-fuel competition which is one of its major drawbacks (Kennes et al. 2016). Feedstocks used in second generation biorefineries are mostly lignocellulosic materials, avoiding such food-fuel competition. However, that raw material is composed of cellulose, hemicelluloses and lignin, and rather complex pre-treatments may often be necessary to obtain simple sugars which can then be metabolized by microorganisms. Besides, lignin does not yield any simple sugars. Therefore, an important amount of lignocellulosic material, cannot be used in the carbohydrate fermentation process (Kennes et al. 2016). The conversion of lignocellulosic biomass or other similar renewable resources into syngas by means of a thermochemical pre-treatment, allows to use the complete feedstock, i.e. cellulose, hemicelluloses but also lignin; to obtain a fermentable gas mixture (Fernández-Naveira et al. 2017a). That syngas can be metabolized by anaerobic bacteria as carbon and energy source to produce a range of fuels (e.g., bioalcohols), chemicals and other bioproducts (e.g., biopolymers) (Abubackar et al. 2011; Bengelsdorf et al. 2013; Lagoa-Costa et al. 2017, Mohammadi et al. 2011). Some industrial emissions are also rich in C1 gases, and sometimes hydrogen, similar as syngas obtained from biomass, and can then also be used by anaerobic bacteria as substrates for their fermentation into alcohols or other commercial products (Kennes and Veiga 2013). Such gas fermentation process needs then to be optimized, trying to determine the best bioreactor operating conditions and avoiding any possible inhibition effect on the bioconversion.

Different parameters affect the efficiency of a fermentation process, either in a positive or in a negative way, and may also affect the nature and concentration of end metabolites. Optimal culture medium composition is one such key parameter. For example, in syngas-metabolizing solventogenic acetogens, the pH of the culture broth as well as the addition or omission of specific trace metals (e.g., tungsten) allow to shift the metabolism towards the production of either acids or alcohols (Abubackar et al. 2015, 2016; Fernández-Naveira et al. 2019). Similarly, the salinity of a fermentation medium can play a role in the activity of microorganisms and should be considered when setting-up a bioprocess. The salinity of a medium may vary, among others during pH adjustment, as the addition of chemicals such as NaOH or HCl will result in the accumulation of salts. The accumulation of salts can switch the bioconversion pattern from solventogenic fermentation to acetogenic (Maddox et al. 1995). In first and second generation production of biofuels and other platform chemicals through sugar fermentation, pH adjustment during pretreatments or fermentation steps may lead to the accumulation of salts or other potentially inhibitory compounds (Casey et al. 2013; Palmqvist and Hahn-Hägerdal 2000). Similarly as in first and second generation processes, pH is a key parameter strongly affecting the metabolism of solventogenic clostridia and acetogens in general, fermenting syngas, while pH adjustment relies on the addition of acids or bases that will ultimately affect salinity. Studies on the effect of high salt concentrations have been done in pathogenic, medical and food-related clostridia mainly. For example, the addition of salt (i.e. sodium chloride) is known to be a common way to prevent the growth of different microorganisms such as *Clostridium* spp. in food products (Lund 1993). It has also been described that the addition of salt at levels of 8.2–10.5% (W/W) allows to prevent spore outgrowth (Khanipour et al. 2014). No study has been reported on the influence of salinity or conductivity of the medium on the activity of syngas-metabolizing strains useful in environmental applications.

In this study, anaerobic syngas fermentation was performed in a stirred tank bioreactor and pH was adjusted after a specific processing time in order to stimulate solventogenesis, i.e. the accumulation of alcohols. Salinity was checked in terms of conductivity during the fermentation process and during pH adjustment. Besides, batch assays were set-up in order to check the inhibitory effect of the salt concentration and conductivity on the *C. carboxidivorans* strain used to inoculate the syngas-fed bioreactor. Conductivity data found in the fermentor were compared to inhibitory values identified from batch assays.

## Materials and methods

### Microorganism and culture media

*Clostridium carboxidivorans* (P7 DSM 15243) was obtained from the Deutsche Sammlung von Mikroorganismen und Zellkulturen GmbH (Braunschweig, Germany), and was maintained anaerobically on modified basal medium (Liou et al. 2005; Tanner 2007) with carbon monoxide (100%) as a carbon source, and at an initial pH of 5.75.

The general composition of the medium used was (per liter distilled water): Yeast extract, 1 g; mineral solution 25 mL; trace metal solution, 10 mL; resazurin, 1 mL; cysteine-HCl, 0.60 g.

The trace metal stock solution was composed of (per liter distilled water): 2 g nitrilotriacetic acid, 1 g manganese sulfate, 0.80 g ferrous ammonium sulfate, 0.20 g cobalt chloride, 0.20 g zinc sulfate, and 20 mg each of cupric chloride, nickel chloride, sodium molybdate, sodium selenate, and sodium tungstate.

The composition of the mineral stock solution was (per liter distilled water): 100 g ammonium chloride, 10 g potassium chloride, 10 g potassium monophosphate, 20 g magnesium sulfate, and 4 g calcium chloride. In the batch assays, different concentrations of sodium chloride were added to each bottle in order to check its effect on the strain. The sodium chloride concentration commonly recommended by culture collections for that strain is 2 g/L.

The composition of the vitamins stock solution added to the batch bottles and the bioreactor was (per liter distilled water): 10 mg pyridoxine, 5 mg each of thiamine, riboflavin, calcium pantothenate, thioctic acid, paraamino benzoic acid, nicotinic acid, and vitamin B_12_, and 2 mg each of d-biotin, folic acid, and 2-mercaptoethanesulfonic acid.

### Bottle batch experiments

Different concentrations of sodium chloride were added in serum vials, per duplicate, in order to check the effect of sodium chloride on bacterial growth and activity. Sodium chloride was added at the following final concentrations: 0.2, 3, 9, 10, 11, 12, 15, 18 and 21 g/L. The medium was introduced in 250 mL anaerobic serum vials with 100 mL working volume. The pH was adjusted to 5.75, and the medium was boiled and flushed with nitrogen to ensure anaerobic conditions. After that, the bottles were sealed with rubber stoppers and aluminum caps and autoclaved for 20 min at 120 °C.

For seeding the medium, 10% biomass in exponential growth phase was inoculated in each bottle with the addition of vitamins at the same concentrations as for growing the inoculum. They were then pressurized to 1.2 bar with 100% CO and were agitated at 150 rpm inside an orbital incubator at 37 °C.

### Bioreactor experiment

The continuous gas-fed bioreactor experiment was carried out in a 2 L BIOFLO 110 bioreactor (New Brunswick Scientific, Edison, NJ, USA) with a working value of 1.2 L. A gas mixture of CO:CO_2_:H_2_:N_2_ (20:20:10:50) was used as a carbon and energy source and was fed at a flow rate of 10 mL/min using a mass flow controller (Aalborg GFC 17, Müllheim, Germany) during all the experiment.

The original basal medium was prepared as described above, with the addition of 2 g/L sodium chloride, and was then autoclaved. After sterilization, the medium was flushed with N_2_ and cooled down to 33 °C. A water jacket allowed to maintain the temperature constant throughout the experiment. During the cooling down process, once the temperature had reached about 35 °C, cysteine-HCl and vitamins were added. Once the bioreactor had reached anaerobic conditions, the N_2_ feed was interrupted and it was then replaced by a syngas mixture fed to the reactor at a flow rate of 10 mL/min, using a microsparger. The agitation speed was maintained constant at 250 rpm. To avoid the formation of vortex in the culture medium, four baffIes were placed inside the bioreactor. The pH and redox potential were constantly measured. The pH was controlled and adjusted to the desired value through the addition of either 1 M NaOH or 1 M HCl solutions. Finally, 10% of the bacterial culture, in exponential growth phase (which was grown with CO as a carbon source during 72 h), was inoculated in the bioreactor.

Initially, during the first experimental period, pH was maintained constant at a value of 5.75, then, when the maximum concentration of acids was reached, the pH was lowered to 4.8 through stepwise addition of HCl over a period of 8 h, in order to avoid any possible inhibition that may result from a sudden pH drop. 170 h after the pH drop, its value was increased up to 5.75 again, adding NaOH gradually. 50 h later, the medium of the reactor was partially replaced. When the maximum concentration of acids was reached again, HCl was added again to decrease the pH to a value of 4.8.

### Growth measurement

For the batch assays in bottles, samples were taken at least twice a day during the exponential growth phase and once a day after that, withdrawing 1 mL of liquid sample from each bottle. The optical density (OD_λ_ = 600 nm) was measured on a UV–visible spectrophotometer (Hitachi, Model U-200, Pacisa & Giralt, Madrid, Spain) in order to estimate the biomass concentration. A biomass calibration curve had previously been plotted, representing biomass concentration vs optical density, with the aim to estimate the biomass concentration (g/L) based on the absorbance readings.

The growth rates (µ), expressed in h^−1^, for each bottle, were calculated during the exponential growth phase using the following equation:

$$\upmu = \left[ {{\text{Ln}}\left( {{\text{N}}_{\text{t}} } \right) - {\text{Ln}}\left( {{\text{N}}_{0} } \right)} \right]/\left( {{\text{t}} - {\text{t}}_{0} } \right)$$

where N_t_ is the cell density (g/L) at time t (expressed in hours) and N_0_ is the cell density at time 0 (t_0_), when the exponential phase started.

Another parameter estimated in that assay was the IC_50_ (concentration of the tested substance that decreases the growth rate by 50%). It is one of the most commonly used parameters in toxicity assays (Leboulanger et al. 2001). The IC_50_ for each concentration of sodium chloride were estimated using a non-linear regression analysis (four parameters sigmoidal) of the concentration of sodium chloride as logarithm versus the percentage of growth inhibition. The percentage of inhibition is calculated considering that the control bottles correspond to 0% inhibition. All the calculations were made using the regression Wizard software (Sigma-Plot 12.5, SPSS Inc.).

### Gas-phase CO concentrations

Similarly as for biomass sampling, samples were taken once or twice a day in bottles assays, depending on the growth phase. 1 mL gas sample was removed from the headspace of each bottle to estimate CO consumption.

The CO concentration in each bottle was measured using an HP 6890 gas chromatograph (GC, Agilent Technologies, Madrid, Spain) equipped with a thermal conductivity detector (TCD) using Helium as a carrier gas. The GC was fitted with a 15 m HP-PLOT Molecular Sieve 5A column (ID: 0.53 mm, film thickness: 50 μm). The oven temperature was maintained constant at 50 °C, while the temperature of the injection port and the detector were maintained constant at 150 °C.

### Fermentation products

In case of the bioreactor studies, 1 mL liquid samples were taken daily to check the nature and concentration of fermentation products on an HPLC (HP1100, Agilent Co., USA) equipped with a supelcogel C-610 column and a UV detector at a wavelength of 210 nm. A 0.1% ortho-phosphoric acid solution was used as a mobile phase at a flow rate of 0.5 mL/min. The column temperature was set at 30 °C. The samples were centrifuged (7000*g*, 3 min) using a benchtop centrifuge (ELMI Skyline ltd CM 70M07) and filtered using a 0.22 µm filter, before analyzing them on the HPLC.

### Conductivity measurement

In the bioreactor studies, conductivity was checked at the beginning of the experiment, at the end and after pH changes, when adding NaOH or HCl. For batch bottles experiments, the conductivity was only checked after inoculation, because those assays did not have any pH control and conductivity remained constant. For the measurements, 1 mL liquid sample was removed from the medium and conductivity was measured with a conductimeter (EUTECH INSTRUMENTS αlpha CON560).

## Results

### Continuous gas-fed bioreactor experiment

The bioreactor was set-up with continuous syngas feed and its performance as well as salinity (conductivity) data were monitored as described hereafter.

#### Bioreactor performance

**Fig. 1:**
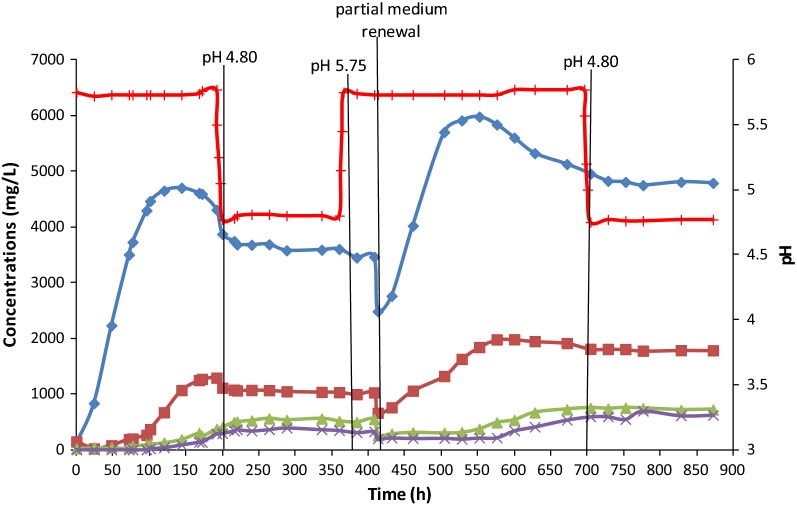
Fermentation products expressed in mg/L over time in continuous gas-fed bioreactor. (Acetic acid represented as filled diamond, butyric acid represented as filled square, ethanol represented as filled triangle and butanol represented as X)

**Fig. 2:**
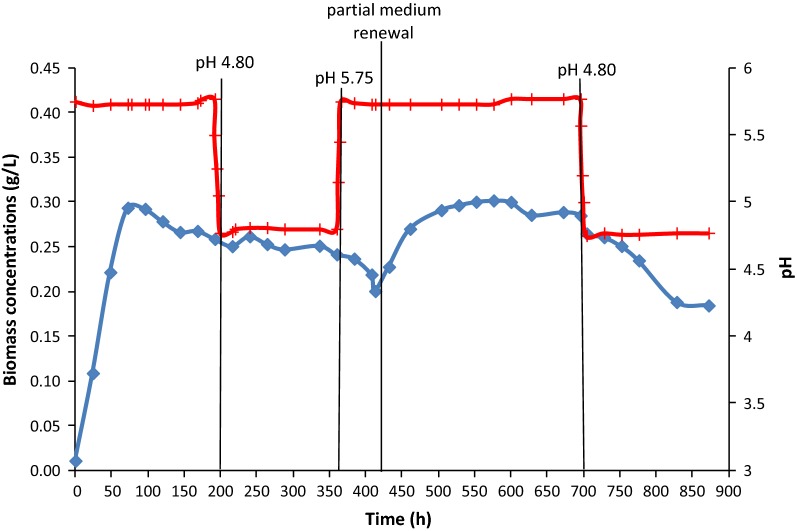
Biomass measurement in g/L over time in gas-fed bioreactor

**Fig. 3:**
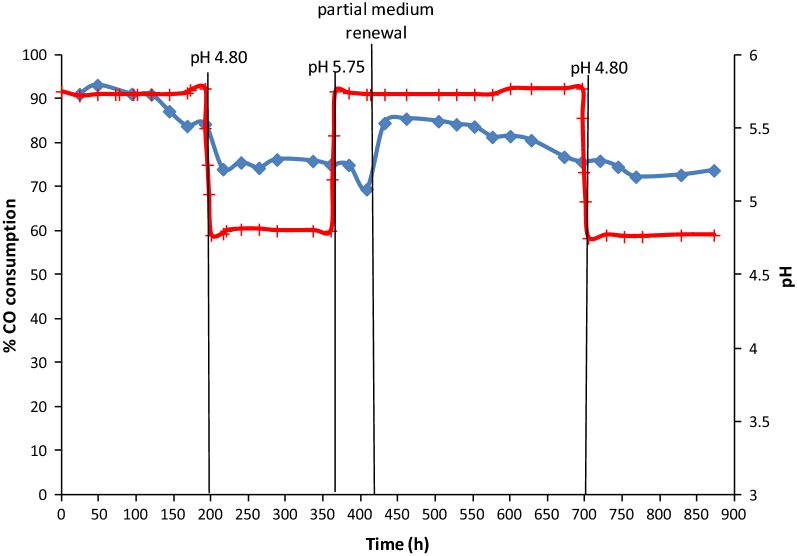
Percentage of CO consumption over time in gas-fed bioreactor

Acetogenesis, with the production of organic acids, started immediately after bioreactor inoculation. The first acid to be produced by *C. carboxidivorans* was acetic acid, which was the only acid detected in large amounts during the first 3 days of operation (Fig. 1). Later, 72 h after inoculation, butyric acid appeared, when acetic acid had already reached a high concentration of 3.50 g/L. The maximum concentrations of these acids were 4.70 g/L for acetic acid, 144 h after inoculation, and 1.30 g/L for butyric acid, 192 h after inoculation. Growth of *C. carboxidivorans* was simultaneous to the production of acetic acid, following a common pattern. A maximum biomass concentration of 0.294 g/L was reached after 72 h (Fig. 2), slightly before the concentration of acetic acid stopped increasing (Fig. 1). The growth rate was calculated in the exponential phase, between 0 and 48 h, yielding a value of 0.063 h^−1^. During that first experimental stage, at pH 5.75, bacterial growth and the production of acids were concomitant to a fast CO consumption, starting soon after inoculation. During the first 120 h, a maximum CO consumption, between 90 and 93%, was observed (Fig. 3). After 120 h, the CO consumption started to decrease, around 83%, at that same pH value of 5.75. Concerning the production of alcohols, some ethanol was first detected, 72 h after inoculation, whereas butanol was not observed until t = 101 h (Fig. 1). The maximum concentrations of ethanol and butanol, before the artificial pH drop (t = 200 h), were 0.40 and 0.29 g/L, respectively.

In order to stimulate the solventogenic phase, artificial acidification down to pH 4.80 was applied at t = 200 h. After the pH change, both acetic and butyric acids started to be consumed; although the acetic acid concentration had also already been found to slightly decrease before lowering the pH value. In Fig. 1, consumption of 0.700 g/L acetic acid and 0.300 g/L butyric acid can be observed during this period. After the pH drop, some hexanoic acid was detected in the medium. However, for technical reasons the concentration of C6 compounds could only be measured later, after the experiment had finalized. Since the exact concentrations detected (corresponding to a few hundreds of mg/L) were not fully reliable, the data have not been plotted in Fig. 1, as this does also not affect the main goal and conclusions of the study, focusing on estimating salt inhibition. In any case, it can be stated that the production of such compound was similar as in other related studies (Fernández-Naveira et al. 2017b, 2019). During the low pH conditions, CO consumption started decreasing down to 74–76% (Fig. 3). Although biomass appeared initially to decay somewhat at pH 4.80, the biomass concentration remained then roughly constant around 0.260 g/L during this experimental stage (Fig. 2). After the artificial pH drop, only 0.155 g/L ethanol and 0.100 g/L butanol were produced and hardly any acids were consumed, although a low pH is expected to be more favourable to solventogenesis than higher pH values. Therefore, it was decided to check if changes in the salt concentration and conductivity, due to the artificial pH drop reached by adding HCl to the medium, could be a possible reason for the low alcohol productivity, slight biomass decay and reduced gas consumption. This was checked in batch assays described in the next section.

Because of the lower bioreactor performance, it was decided to increase the pH again to 5.75. However, no significant improvement in acids consumption or production was observed (Fig. 1), while the biomass concentration kept decreasing slowly (Fig. 2). Therefore, a partial medium replacement was decided and part of the bioreactor’s fermented medium was replaced by fresh medium, to check if any inhibitory medium component may have accumulated, including salts, or if some key compound may have become limiting. After the partial medium renewal, biomass started immediately to grow again, reaching a maximum value of 0.30 g/L, at t = 528 h (Fig. 2), which is similar to the original highest steady-state cell concentration reached in the first stage of the experiment. It can also be observed, in Fig. 1, how the partial medium renewal exerted a positive effect on the production of acids, with a fast increase of their concentrations up to maximum values of 6.00 g/L for acetic acid (t = 552 h) and 1.98 g/L for butyric acid (t = 600 h). As a consequence of the bacterial growth and acids production, CO consumption recovered as well, reaching a maximum value of 85%. Then, later on, CO consumption started to decrease again, around t = 600 h, when the maximum concentration of acids was reached. With this partial medium renewal, the production of alcohols was also stimulated, reaching maximum concentrations of ethanol and butanol for that second period of 0.750 and 0.600 g/L, respectively, at t = 700 h (Fig. 1).

Subsequently, in the last experimental period, after t = 700 h, HCl was gradually added in order to decrease the pH value to 4.80 again, aiming at stimulating solventogenesis. A slow conversion of acids had already taken place at pH 5.75, while the pH decrease seemed to inhibit any further alcohols production. As observed in Fig. 1 no more alcohols were produced after the pH was modified. A dramatic decrease of the biomass concentration (Fig. 2) and slowdown of gas consumption (Fig. 3) were also found and the experiment was then stopped.

The total net production of alcohols was analysed, reaching the following values: 1.05 g/L of ethanol, corresponding to 0.56 g/L produced between t = 0 h and t = 264 h and 0.49 g/L between t = 413 h and t = 704 h. In both cases such production took mainly place at pH 5.75. There was no production of acids either at low pH although this was expected as a low pH might stimulate the production of alcohols through acids consumption, but would not favor the production of such acids. In the case of butanol, an overall production of 0.89 g/L was observed, with 0.39 g/L produced during the first pH 5.75 period, between t = 101 h and t = 288 h, and 0.50 g/L during the second pH 5.75 period, between t = 576 h and t = 776 h, in a similar way as for ethanol. That way, total amounts of 1.26 g ethanol and 1.07 g butanol had accumulated in the 1.2 L bioreactor.

#### Salinity effect and conductivity measurements

Several hypotheses were considered in order to try to clarify the problem with the solventogenic stage. Inhibition could have been provoked by the salinity due to the addition of NaOH and HCl required for the artificial pH decrease. On the other side, *C. carboxidivorans* might be sensitive to the pH changes and the way they were applied in the present study, and the artificial pH decrease down to pH 4.80 might not be a fully suitable strategy to stimulate solventogenesis in this case. Finally, a combination of artificial acidification, low pH and salinity, and may be other conditions, might altogether have affected the bacterial solventogenic activity.

At different stages of the fermentation process described above, samples were taken in order to check the conductivity of the culture medium and estimate a possible relationship between salinity and inhibitory conditions. The following values were obtained: 13.85 mS/cm at t = 0 h, on starting the experiment; 14.59 mS/cm after the first pH drop, at t = 240 h; 17.33 mS/cm after the pH increase and before the partial medium replacement, at t = 384 h; 15.30 mS/cm after the partial medium replacement (t = 432 h); and 16.21 mS/cm at the end of the experiment.

Since fluctuations of conductivity were observed during the different experimental periods, batch bottle inhibition experiments were set-up using media with different salt additions and conductivities, as described hereafter.

### Batch bottle experiments and effect of salinity

Batch assays were performed in presence of different salt concentrations, and thus conductivities, to evaluate their effect of the growth and activity of *C. carboxidivorans*.

#### Growth and biomass concentration

**Fig. 4:**
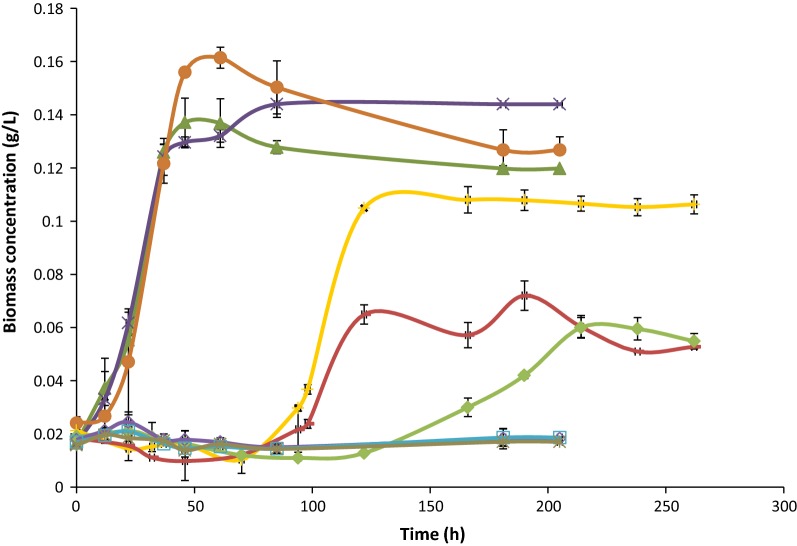
Measured biomass concentration expressed as g/L over time. (0.2 g/L of sodium chloride represented as filled triangle, 3 g/L of sodium chloride represented as X, 9 g/L of sodium chloride represented as filled circle, 10 g/L of sodium chloride represented as +, 11 g/L of sodium chloride represented as −, 12 g/L of sodium chloride represented as filled diamond, 15 g/L of sodium chloride represented as open diamond, 18 g/L of sodium chloride represented as open square and 21 g/L of sodium chloride represented as *

Three different growth patterns can be described, depending on the sodium chloride concentration, i.e. conductivity (Fig. 4). In media with concentrations between 0.2 and 3 g/L sodium chloride, *C. carboxidivorans* started growing soon upon inoculation of the bottles, without any significant lag phase, and reached similar maximum biomass concentrations of 0.140 g/L after 46 h in 0.2 g/L bottles, and 0.144 g/L after 85 h in 3 g/L bottles. The growth pattern observed in the bottles with 9 g/L sodium chloride can still be considered to be similar as in the other assays at the concentrations described above (up to 3 g/L), with the only difference that in this case *C. carboxidivorans* showed a slightly longer lag phase of 22 h, reaching a maximum biomass value of 0.161 g/L after 61 h, which is close to the values obtained with the previous concentrations assayed. The second pattern is for sodium chloride concentrations ranging between 10 and 12 g/L, in which inhibitory effects started to be observed, and *C. carboxidivorans* exhibited a lag phase of about 100 h after inoculation, or even 122 h at the highest concentration of 12 g/L. Although the 10 g/L assay still reached a maximum biomass accumulation of 0.108 g/L, after 166 h, those values were lower in the other two cases. At 11 g/L sodium chloride, the biomass concentration was only 0.072 g/L after 190 h; and that parameter reached 0.060 g/L, after 240 h, at a salt concentration of 12 g/L. The third pattern corresponds to bottles with still higher concentrations of sodium chloride, of 15, 18 and 21 g/L. In those bottles, complete inhibition was found and the highest amount biomass in those experiments did not exceed 0.003 g/L. That way, as can be seen in Fig. 4, increasing the concentration of sodium chloride in the fermentative broth, results in a negative effect on the growth pattern of *C. carboxidivorans* when surpassing a given threshold salt level.

#### Specific growth rates

The specific growth rates were estimated for each sodium chloride concentration used in this study (Table 1). A negative effect on the growth rate of *C. carboxidivorans* could be observed at increasing sodium chloride concentrations above a given threshold value. Up to nearly 9 g/L sodium chloride, no clear inhibition was detectable, although inhibition seems then to start at values around 9 g/L and higher. The slight variations in growth rates over that concentration range of 0–9 g/L could be related with the sampling time or with the state of the inoculum or even with the experimental accuracy, but otherwise no significant differences were observed. However, at higher sodium chloride concentrations, both the growth rates and maximum biomass concentrations decreased, even observing complete inhibition of *C. carboxidivorans* when the sodium chloride concentration reached 15 to 21 g/L (Table 1).

**Table 1:** Maximum specific growth rates for each sodium chloride concentration, expressed in h^−1^ and sodium chloride concentration represented as conductivity in mS/cm

Sodium chloride concentration (g/L)	Maximum specific growth rate (µ) (h^−1^)	Conductivity (mS/cm)
0.2	0.055	10.0
3	0.055	17.1
9	0.052	26.4
10	0.044	27.1
11	0.032	29.9
12	0.021	32.8
15	0	37.9
18	0	43.6
21	0	48.0

#### Half maximal inhibitory concentration (IC_50_)

An important parameter in toxicity assays is the IC_50_. To check how restrictive the salinity of the fermentation broth could be on the activity of *C. carboxidivorans*, the IC_50_ values were estimated 181 and 202 h after inoculation. The values obtained were 10.79 g/L and 11.02 g/L, respectively. Those values correspond to the concentration at which inhibitory effects started being detected in the experiment, as shown in Fig. 4, where no inhibition was found between 0.2 and 9 g/L, while longer lag phases and reduced maximum biomass concentrations were observed at concentrations exceeding 9 g/L.

#### Conductivity measurement

When gas fermentation experiments are carried out with *C. carboxidivorans* to obtain alcohols, non negligible amounts of NaOH and HCl are consumed during the acetogenic and solventogenic stages in order to adjust the pH. The addition of those two chemicals would change the salinity and conductivity of the fermentation broth. As a result, this could have a negative effect on the bioconversion process. For each sodium chloride concentration assayed, the corresponding conductivity was measured (Table 1), in order to estimate the conductivity range that would cause a negative effect on *C. carboxidivorans*, and eventually complete inhibition. With these values, and the data obtained in the IC_50_ analysis (10.79 and 11.02 g/L of sodium chloride), the value of conductivity which would cause 50% inhibition on the growth of *C. carboxidivorans* was around 29.9 mS/cm.

The medium used in the continuous gas-fed bioreactor experiment described here, and in similar experiments with *C. carboxidivorans* and syngas as a carbon source described in recent literature and the initial sodium chloride concentration (Fernández-Naveira et al. 2016a, 2017b) correspond to the composition commonly recommended in the standard culture medium (2 g/L sodium chloride). This corresponds to an initial conductivity around 14 mS/cm. At the end of the bioreactor experiment, after consumption of NaOH and HCl, the conductivity of the fermentation broth was 16–18.7 mS/cm. These values observed in the bioreactors are far from the IC_50_. Therefore, the conductivities reached in the fermentation broth due to the addition of NaOH and HCl should not represent a problem for the bioconversion process and no inhibition should be expected in this specific study; although other conditions where different concentrations of NaOH or HCl or other compounds are added during the process might eventually reach inhibitory values, which should then be taken into account.

#### CO consumption

**Fig. 5:**
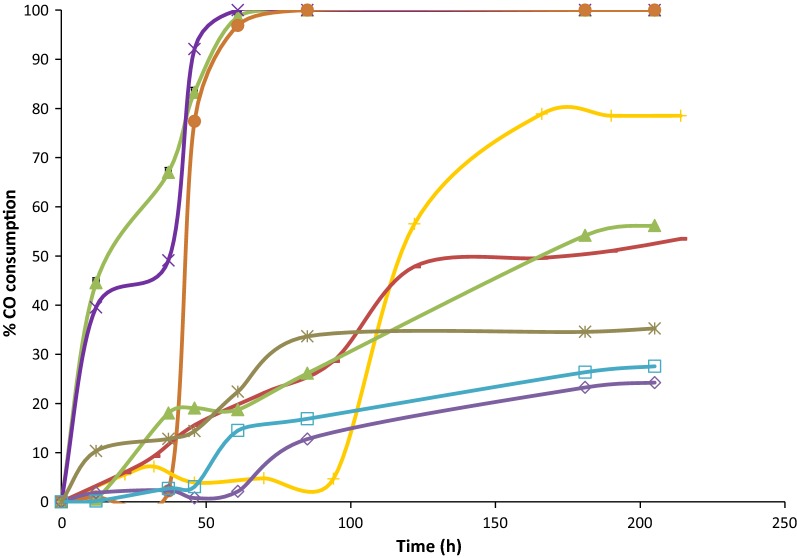
Percentage of carbon monoxide consumption over time. (0.2 g/L of sodium chloride represented as filled triangle, 3 g/L of sodium chloride represented as X, 9 g/L of sodium chloride represented as filled circle, 10 g/L of sodium chloride represented as +, 11 g/L of sodium chloride represented as −, 12 g/L of sodium chloride represented as filled diamond, 15 g/L of sodium chloride represented as open triangle, 18 g/L of sodium chloride represented as open square and 21 g/L of sodium chloride represented as *

Similarly as observed for the biomass concentration, three patterns were also observed for the percentage CO consumption (Fig. 5). Similar fast CO consumptions were typical at sodium chloride concentrations between 0.2 and nearly 9 g/L (Fig. 5). In all those bottles, except at 9 g/L, CO started to be consumed soon after inoculation, reaching 100% consumption quite fast and exhausting the carbon source 61 h after starting the experiment. In the case of bottles with 9 g/L sodium chloride, the consumption of CO started a few hours later (12 h) corresponding also to the moment when growth started. A second pattern can be observed for concentrations between 10 and 12 g/L sodium chloride. In those bottles, CO consumption was slower and the substrate did not get fully exhausted. The maximum CO consumptions were 79% after 166 h, 53.5% after 214 h, and 56% after 204 h for the bottles with 10, 11 and 12 g/L, respectively (Fig. 5). The lower CO consumption in those bottles is also related with the lower biomass concentration obtained in comparison with the first set of assays at lower sodium chloride concentrations. For the last group of bottles, corresponding to 15, 18 and 21 g/L sodium chloride, still lower CO consumptions were observed with maximum values of 24%, 27.6% and 35% after 204 h for each concentration. In those bottles no significant growth was detected, and the biomass concentration remained low though constant during all the experimental process. That way some consumption of CO was observed which may be reasonable as it could have been used for biomass maintenance or to satisfy other metabolic needs. Some minimal loss during the sampling can also not completely be ruled out.

## Discussion

*Clostridium carboxidivorans* typically produces acids and alcohols using syngas as a carbon and energy source. Under given conditions, the fermentation pattern is well reproducible with production of acids first, at high or near neutral pH, followed by the production of alcohols at lower pH (Fernández-Naveira et al. 2016a, 2017b). The pattern observed here in the bioreactor experiments agrees with data reported recently under similar conditions (Fernández-Naveira et al. 2017b). Besides, as expected, the growth of *C. carboxidivorans* was simultaneous to the production of acids, mainly acetic acid (Fernández-Naveira et al. 2016a, 2017b). The values of maximum biomass concentration and growth rate are also similar to values reported in other studies (Fernández-Naveira et al. 2017b). If some of the experimental conditions are modified the growth pattern and/or production of metabolites may vary, e.g., in the presence of specific trace metals or at different pH values (Fernández-Naveira et al. 2019).

A low pH stimulates the solventogenic stage and accumulation of alcohols, after the production of acids (Fernández-Naveira et al. 2017a, b). pH decrease may either be natural, as a result of the production of acids, or pH may be forced to decrease faster, earlier, or to lower values, if needed, through artificial acidification. However, the artificial acidification applied here, through the addition of HCl, seemed to have a negative effect on the process. The same was observed in other experiments in our laboratory in which clostridia (e.g., *C. carboxidivorans*, *C. autoethanogenum*) seemed to tolerate better natural rather than artificial acidification. Artificial pH changes result in increased salinity, not occurring with natural acidification and, this may affect bacterial activity.

In the batch assays, the maximum biomass concentrations and growth rates found under non-inhibitory conditions (normal medium with no addition of sodium chloride and no increase in conductivity) were in the same range as observed in other studies, with only small variations which can be related to the experimental conditions, e.g., pH, temperature, medium (Fernández-Naveira et al. 2016a, b). Hardly any research has been reported on the effect of salinity on clostridia and acetogens used in environmental applications, while published studies are available on pathogenic clostridia or strains typically found in food research. A strain of *C. botulinum* 62A was reported to tolerate sodium chloride concentrations up to 3% without any inhibition (Montville 1983). Similarly, the parent strain of *C. botulinum* ATCC 3502 with a growth rate of 0.31 h^−1^ in presence of 1% sodium chloride, was still able to grow in presence of 4% sodium chloride, but with a quite lower growth rate of 0.10 h^−1^ (Derman et al. 2015). For other bacteria such as *C. tyrobutyricum* different strains found in dairy products tolerated at least 3% salt in milk, but complete inhibition was often already observed at 3.5% (Ruusunen et al. 2012). In cooked ham and beef, among three *C. perfringens* strains, all could develop at sodium chloride concentrations up to 2% but complete inhibition was generally found above 3% salt (Zaika 2003). Quite less information is available on strains of interest in biorefineries. *C. acetobutylicum* can convert carbohydrates from renewable sources into solvents such as acetone, butanol, and ethanol (ABE fermentation) (Kennes et al. 2016). That species seems to face total inhibition with 30 g/L sodium chloride or 45 g/L magnesium chloride (Maddox et al. 1995). Many of the above mentioned bacteria can generally still grow in presence of about 2–4% sodium chloride, depending on the strain, while *C. carboxidivorans* appears to be completely inhibited at such concentrations. An IC_50_ of 15 g/L sodium chloride was reported for *C. acetobutylicum* (Maddox et al. 1995). In comparison with the data obtained for *C. carboxidivorans* in the present study, *C. acetobutylicum* showed to be somewhat more resistant to high salinity than the *C. carboxidivorans* strain used in this work, with an IC_50_ around 11 g/L for sodium chloride.

The salinity (conductivity) of the medium (e.g., sodium chloride concentration) can affect the internal functions of the cell, causing reduction of the performance of the process (e.g., anaerobic gas fermentation) at high salt concentrations. Although the salt concentration in the bioreactor was below the inhibitory values identified for *C. carboxidivorans* in batch assays, that strain seems to be more sensitive to salinity than previously described clostridia. However, the salt concentrations found in this syngas fermentation process are below highly inhibitory values. Therefore, it should not significantly have affected the long term efficiency of the bioconversion. The results show that concentrations below 9 g/L do not affect the strain’s activity while concentrations of 15 g/L or higher do completely inhibit *C. carboxidivorans*, with intermediate inhibitory effects at salt concentrations between 9 and 15 g/L. Since conductivity did not seem to affect the stability of the process and the low conversion of acids in this study, the pH value could have played a role. Indeed, the optimal pH range for that strain has been suggested to be 5–7 (Liou et al. 2005; Fernández-Naveira et al. 2017c) and a pH around 4.80,—although not reported to be inhibitory—, can reasonably be assumed to be close to a lower threshold value that could have avoided the total conversion of acids into alcohols. Besides, in another recent study it was found that limiting the pH drop to a minimum value of 5.0, instead of 4.80, does allow the near complete conversion of acids (e.g., acetic acid) into alcohols (e.g., ethanol) in *C. carboxidivorans* (Fernández-Naveira et al. 2019). In any case, a natural acidification process, without any addition of acid or base, may be less stressful for the bacteria, than artificial acidification, and thus allow a better conversion of acids into alcohols.

It can be concluded that: (a) salinity below 9 g/L sodium chloride does not cause any inhibition on *C. carboxidivorans*, (b) concentrations between 9 and 15 g/L show partial inhibition, (c) concentrations higher than 15 g/L have a significant negative effect on *C. carboxidivorans*, (d) IC_50_ values were between 10.79 and 11.02 g/L, (e) the maximum salinity measured in the syngas fermentation broth was lower than the IC_50_ values determined in batch assays.

## Data Availability

Can be obtained from the corresponding author.
